# Bronchopulmonary lophomoniasis: emerging disease or unsubstantiated legend?

**DOI:** 10.1186/1756-3305-7-284

**Published:** 2014-06-23

**Authors:** Rafael Martínez-Girón, Hugo Cornelis van Woerden

**Affiliations:** 1 Protozoal Respiratory Pathology Research Unit, INCLÍNICA Foundation, Calvo Sotelo, 16-3° dcha, 33007 Oviedo, Spain; 2 Institute of Primary Care & Public Health, Cardiff University School of Medicine, Neuadd Meirionnydd, Heath Park, Cardiff CF14 4YSUK

**Keywords:** Bronchopulmonary lophomoniasis, *Lophomonas blattarum*, Cockroaches

## Abstract

The relationship between *Lophomonas*, a genus of multiflagellated protozoa, and respiratory pathology has recently received attention. Here, we summarize the origin, mode of transmission, pathogenic mechanism and relevant clinical data of bronchopulmonary lophomoniasis.

## Headings

The relationship between *Lophomonas*, a genus of multiflagellated protozoa, and respiratory pathology has recently received attention in a Chinese article by Mu *et al*. [1] which concludes that, “In the past 20 years, all the diagnosed cases as pulmonary *Lophomonas blattarum* infection reported in our country were misdiagnosed. Currently, there is no evidence to show *Lophomonas blattarum* as a pathogen resulting in pulmonary infection.” This conclusion is at odds with a literature review in which we identified 61 case reports of pulmonary *L. blattarum*[2].

We recognise that it is possible for “detached ciliary tufts” (also described as “Ciliocytophthoria” phenomena) to be confused with protozoa in sputum samples. Cytoplasmic remnants with cilia are a frequent finding in the bronchial secretions from patients with obstructive pulmonary disease and asthma [3] Nevertheless, parasitic infection caused by the multiflagalled protozoon *L. blattarum* has been described [4], and its presence in respiratory secretions, in both fresh and stained smears, has been observed [5].

**Figure 1:**
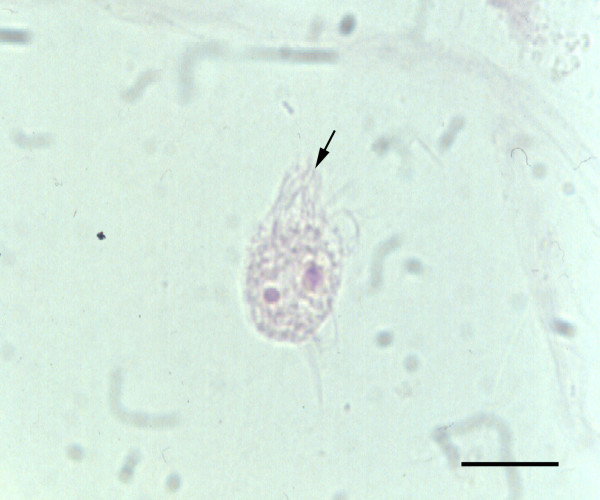
***Lophomonas blattarum *in a sputum smear.** Note the typical arrangement to the flagella in two pairs of tufts (black arrow). Into the granular cytoplasm it is possible to observe two red phagocyted bodies (Papanicolaou stain, × 1000. Scale bar = 30 μm).

*L. blattarum* was first described by S. Stein in 1860, from the gut of the cockroach Blatta orientalis [6]. It was, also observed in the hindgut of the other cockroaches such as *Periplaneta americana* and *Blatella germanica*. A detailed morphologic description of *L. blattarum* observed under a light microscope was undertaken by Brugerolle and Lee [7]. The main features described were: round to ovoid in shape (20–60 μm in diameter), a double tuft of flagella inserted at the anterior end, and a certain plasticity of the cytoplasm, containing coarse granules and some phagocytic vacuoles (Figure 1), whilst on most occasions, the nucleus was not visible.

It is well recognised that certain protozoa are capable of forming protective cysts to survive in difficult conditions. The encystment of *L. blattarum* has been illustrated in detail in stained preparations by Kudo [8]. It is reasonable to hypothesise that viable cysts of *L. blattarum* could enter into the human body by inhalation or ingestion of material contaminated by cockroach faeces. Once in the bronchial tree, and under favourable conditions of temperature and humidity, excystation would logically be expected. Thus, and as it happens with other types of human enteric protozoa such as *Entamoeba histolytica*, *Balantidium coli*, *Toxoplasma gondii*, *Cryptosporidium parvum* and *Cyclospora cayetanensis*[9,10], the cockroaches may also be regarded as vectors for this multiflagellated protozoon.

As regard its pathogenic mechanism, it is well-known that certain strong protozoan proteases are involved in different mechanisms of cellular damage such as cytoadherence, breakdown of epithelial barriers, induction to apoptosis, etc. Recently, *Acanthamoeba* protease activity has been reported to have induced allergic airway inflammation in mice via PARs-2 [11]. In a similar context, *L. blattarum* could damage the respiratory epithelium.

A spectrum of clinical manifestations of infestation with *L. blattarum* can be described, ranging from mild cough and slight wheezing to severe respiratory insufficiency with purulent exudates, high fever and radiological signs of pulmonary consolidation. The diagnosis may be confirmed by observing living protozoal forms in fresh samples from respiratory secretions (sputa, bronchial aspirates and bronchoalveolar lavages), where the characteristic irregular movement of flagella can be observed. Alternatively, identification can be undertaken by the staining of smears using Mallory’s trichromic stain or a combination of Papanicolaou and Giemsa stains.

Metronidazole and its derivatives have been utilized for the treatment of this pulmonary infestation in a number of case reports with satisfactory outcomes.

We believe that the observation under light microscopy of this multiflagellated protozoon in symptomatic patients, who respond positively to antiprotozoal therapy, can reasonably be described as bronchopulmonary lophomoniasis. It is possible that the organism exists as a commensal in some individuals and may only be pathogenic in the context of a causal web of aetiological factors [12]. It is also possible that more than one species is being observed which has similar features under light microscopy. However, we believe that this infection should be recognised as a potentially important emerging field of study within respiratory medicine. The development of a technique to culture the organism or the use of molecular techniques is required to resolve the issue.

## Competing interests

The authors declare that they have no competing interests.

## Authors’ contributions

RM wrote the initial draft. HC revised the manuscript and suggested improvements. Both authors read and approved the final version of the manuscript.
